# Add-On Effect of Chinese Herbal Medicine on Mortality in Myocardial Infarction: Systematic Review and Meta-Analysis of Randomized Controlled Trials

**DOI:** 10.1155/2013/675906

**Published:** 2013-01-10

**Authors:** Vincent C. H. Chung, Mao Chen, Qin Ying, Wilson W. S. Tam, Xin Yin Wu, Polly H. X. Ma, Eric T. C. Ziea, Vivian C. W. Wong, Jin Ling Tang

**Affiliations:** ^1^The Jockey Club School of Public Health and Primary Care, The Chinese University of Hong Kong, Hong Kong; ^2^Chinese Medicine Department, Hong Kong Hospital Authority, Hong Kong

## Abstract

In China, Chinese herbal medicine (CHM) is widely used as an adjunct to biomedicine (BM) in treating myocardial infarction (MI). This meta-analysis of RCTs evaluated the efficacy of combined CHM-BM in the treatment of MI, compared to BM alone. Sixty-five RCTs (12,022 patients) of moderate quality were identified. 6,036 patients were given CHM plus BM, and 5,986 patients used BM only. Combined results showed clear additional effect of CHM-BM treatment in reducing all-cause mortality (relative risk reduction (RRR) = 37%, 95% CI = 28%–45%, *I* ^2^ = 0.0%) and mortality of cardiac origin (RRR = 39%, 95% CI = 22%–52%, *I* ^2^ = 22.8). Benefits remained after random-effect trim and fill adjustment for publication bias (adjusted RRR for all-cause mortality = 29%, 95% CI = 16%–40%; adjusted RRR for cardiac death = 32%, 95% CI = 15%–46%). CHM is also found to be efficacious in lowering the risk of fatal and nonfatal cardiogenic shock, cardiac arrhythmia, myocardial reinfarction, heart failure, angina, and occurrence of total heart events. In conclusion, addition of CHM is very likely to be able to improve survival of MI patients who are already receiving BM. Further confirmatory evaluation via large blinded randomized trials is warranted.

## 1. Background 

### 1.1. Myocardial Infarction: Disease Burden and Therapeutic Options

Incoronary artery disease, a critical reduction of the blood supply to the heart may result in myocardial infarction (MI), a phenomenon owing to the formation of an area of necrosis in heart muscles caused by inadequate supply of blood to the muscles, usually as a result of occlusion of a coronary artery. About a quarter of MI patients will die from it due to complications including cardiogenic shock, cardiac perforation, embolism, heart failure, papillary muscle rapture, rhythm disturbances, or autoimmune pericarditis. Current evidence on biomedicine (BM) treatment suggests that aspirin, thrombolytics with or without adding low-molecular-weight heparin, beta-blockers, ACE inhibitors, and nitrates are beneficial for improving outcomes in people with MI. Invasive procedures including coronary artery bypass grafting (CABG) and percutaneous transluminal coronary angioplasty (PTCA, balloon angioplasty) were also found to be useful. However, their efficacy in preventing death is not without limitations. For instance, beta-blockers have no short-term effect on mortality, and they may increase the risk of cardiogenic shock. Thrombolytics may cause stroke and major bleeding while reducing mortality, and those who are treated will receive no additional benefits from nitrates [1]. 

Despite these therapeutic advances, coronary artery disease remained to be the foremost leading cause of death in both low- and middle income countries as well as high-income countries, contributed 11.8% and 17.3% of total deaths, respectively [2]. Researchers are evaluating the potential benefits and harms of add-on treatments like vasodilators and positive inotropes on mortality [3]. Chinese herbal medicine (CHM) is another novel candidate as an add-on treatment. 

### 1.2. Chinese Herbal Medicine for Treating Myocardial Infarction

In China, CHM is widely prescribed in both outpatient and inpatient settings [4]. Amongst community health clinics, 75% provide both BM and traditional Chinese medicine (TCM) treatments. TCM hospitals comprised 13.8% of all hospitals, and 90% of the BM hospitals are annexed with TCM departments [5]. Given the omnipresence of TCM services within the Chinese healthcare system, it is not uncommon for clinicians to prescribe CHM as an adjunct to BM treatment in the management of potentially life-threatening conditions including MI [6]. One of the most researched single herbs is *Radix Astragali, *which exerts its therapeutic effectiveness by inhibiting cardiac fibrosis, reducing infarct size, and increasing capillary and arteriole densities [7]. Commonly used Chinese proprietary medicines include Shexiangbaoxin tablets and Tongxinluo capsules. Shexiangbaoxin tablets are found to slow MI pathogenesis by inhibiting hypertrophy related metabolites [8]. On the other hand, Tongxinluo capsules act by promoting local blood supply and thus limit infarct size [9]. CHM injections based on sheng mai san are also widely prescribed. It reduces infarct size via the activation of protein kinase C, opening of the mitochondrial KATP channels, and lowering the concentration of 5-hydroxytryptamine, norepinephrine, methionine-enkephalin, and leucine-enkephalin [10, 11]. 

### 1.3. Synthesizing Chinese Herbal Medicine Trials: Focusing on Objective Outcomes

The average effect of these CHM formulae as an adjunct to BM could be estimated using random effect meta-analyses of randomized controlled trials (RCTs) [12]. One of the major caveats in conducting systematic reviews on CHM is that existing RCTs are often prone to high risks of bias, thus limiting their usefulness in elucidating treatment effectiveness [13]. However, results from a recent metaepidemiological study have provided an alternative perspective on this issue. It is suggested that objective outcomes are less susceptible to bias associated with inadequate allocation concealment and blinding [14, 15]. Accordingly, by focusing on objective outcomes like mortality, we may partially overcome limitations imposed by the relatively high risk of bias amongst CHM trials. 

### 1.4. Aim of This Paper

Taking into account the methodological considerations above, we performed a systematic review and meta-analysis of RCTs on the efficacy and safety of CHM for MI as an add-on to BM treatment, with a focus on objective critical outcomes including death, recurrent myocardial infarction, and other post-MI cardiac consequences.

## 2. Methods

### 2.1. Criteria for Considering Studies for This Paper

We included RCTs comparing the efficacy and safety of CHM plus BM versus BM alone. CHM is defined as any preparation containing at least one herb or its extraction referenced in the 2010 Chinese Pharmacopeia [16]. We included RCTs which enrolled adult MI patients regardless of gender, age, ethnicity, or comorbidities. We focused on the primary outcomes of (i) mortality of cardiac origin and (ii) all-cause morality. We also consider the following as secondary outcomes: (i) recurrence of MI and (ii) other nonfatal, post-MI cardiac outcomes including cardiac arrhythmia, heart failure, cardiac rupture, cardiogenic shock, and angina. Adverse events reported by authors were also summarized. We imposed no restrictions on language and publication status. 

### 2.2. Search Methods for Identification of Studies

**Figure 1:**
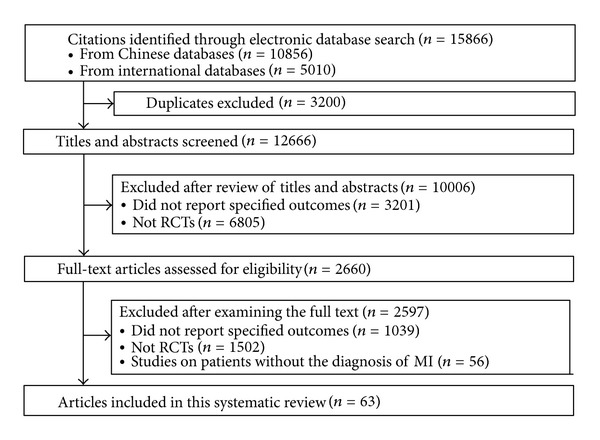
Flow chart of literature search and study selection.

We searched 8 electronic databases since their inception to July 2010, including CENTRAL, MEDLINE, EMBASE, CINAHL, AMED, Chinese Biomedical Database (CBM), Chinese Medical Current Contents (CMCC), and Traditional Chinese Medical Literature Analysis and Retrieval System (TCMLARS) (Figure 1). Search strategies are shown in Appendix 1 in the Supplementary Materials available online at http://dx.doi.org/10.1155/2013/675906.

### 2.3. Data Collection and Analysis

#### 2.3.1. Selection of Studies, Data Extraction, and Risk of Bias Assessment

Two reviewers (Y. Qin and C. Mao) independently screened the titles and abstracts to assess their eligibility. Full texts of potentially eligible citations were retrieved for detailed examination. Selection discrepancies were settled through discussions between these two authors. The remaining disagreements were resolved by consulting another author (J. L. Tang). For included RCTs, comprehensive information on patients, CHM interventions, and baseline and control treatments, as well as outcomes, was extracted. Risks of bias amongst included RCTs were evaluated by the Cochrane collaboration's risk of bias assessment tool [17]. The assessment composed of a description and a judgement for each entry in a risk of bias table, including (i) sequence generation, (ii) allocation sequence concealment, (iii) incomplete outcome data, (iv) selective outcome reporting, and (v) other potential sources of bias. Blinding was assessed for the primary outcome of all-cause morality. 

#### 2.3.2. Data Analysis

Analyses were conducted using Stata 11 and R software. Dichotomous efficacy outcomes were expressed as relative risk reduction (RRR) and relative risk (RR), while RR was used for adverse events. 95% confidence intervals (CIs) were calculated for all estimates. We performed random-effect meta-analysis separately for each outcome. For primary outcomes of all-cause mortality and cardiac death, funnel plots were drawn for assessing publication bias. In case of asymmetry, random trim and fill analysis were performed as a sensitivity analysis [18]. Tests for heterogeneity were performed with chi-squared testes, at a significance level of *P* = 0.1. *I* ^2^ statistic was calculated to estimate variation across studies. We regarded *I* ^2^ < 25% as an indicator of low heterogeneity level, 25–50% as moderate level, and higher than 50% as high level [19]. Heterogeneity was explored with random-effect metaregression using baseline risk, mean age, route of drug administration (oral versus intravenous), and treatment duration as covariates, taking into account the sample size requirement of including not more than 1 covariate for every 10 studies [20]. We expected that higher baseline risk and mean age could be associated with a smaller effect [1], while intravenous administration and longer treatment duration could be associated with a larger effect.

## 3. Results

### 3.1. Literature Search

As shown in Figure 1, our search in electronic bibliographical databases yielded 12,666 citations after removal of duplications, of which 2,660 were classified as potentially relevant and were subjected to a full-text assessment. A total of 65 RCTs published in 63 articles met the inclusion criteria. Details of these studies are presented in Table 1.

### 3.2. Study Characteristics

A total of 6,036 patients were enrolled in the CHM plus BM group, and 5,986 patients were allocated to the BM only group. The average size of the trials was 185 participants (ranging from 28 to 2735 participants per trial). Fifty trials reported treatment duration and the average duration was 68.9 days, ranging from 3 to 1440 days. Forty-nine trials reported the length of followup. The average follow-up length was 7.1 months, ranging from 0.1 to 84 months.

For diagnostic criteria, 36 (55.4%) studies applied the 1979 World Health Organization criteria, which enrolled patients with at least two of the following three presentations: chest pain or discomfort, an elevation in CK-MB levels, or an ECG with significant ST-segment elevations [84]. Four adopted criteria from the Chinese Society of Cardiology [85] and one used criteria from the European Society of Cardiology [86]. Twelve applied author-defined diagnostic criteria, and the remaining 12 did not report criteria used. 

Thirty-one standardized Chinese herbal formulae were examined in 63 (96.9%) of the 65 included studies, while the other two studies used an individualized approach. 32 (50.0%) preparations were administrated orally, 30 (46.9%) were prescribed as herbal injections, and 2 (3.12%) trials used both intravenous and oral treatments. Eight formulae were evaluated by three or more trials. In total, these formulae were assessed in 38 studies, constituting 58.5% of all included trials.

- Nine (13.8%) trials studied Shenmai injection, which contains ginsenoside, ginseng polysaccharide, Ophiopogon polysaccharides, and Ophiopogon flavonoids extracted from* Panax ginseng *and *Ophiopogon japonicas*.
- Five (7.7%) evaluated Huangqi injection manufactured by extracting astragalosides from* Radix Astragali*.
- Another five (7.7%) assessed Shexiangbaoxin tablets, which consisted of Moschus, *Radix Ginseng*, *Borneolum Syntheticum*, *Venenum Bufonis*, *Cortex Cinnamomi*, *Calculus Bovis*, and Styrax.
- Four (6.2%) tested Shengmai injection, which is a mixture of extracts from* Panax ginseng*, *Radix Ophiopogonis*, and *Schisandra chinensis Baill. *
- Another four (6.2%) evaluated Tongxinluo capsules, consisting of *Radix Ginseng, *Scorpio*, Hirudo,* Eupolyphaga *seu Steleophaga, Scolopendra, Periostracum Cicadae, Radix Paeoniae Rubra, *and* Borneolum Syntheticum. *
- Three trials (4.6%) assessed Shenfu injection, which contains Ginsenoside and Aconitine extracted from *Panax ginseng and Aconitum carmichaelii. *
- Another three evaluated Suxiao jiuxin pill (4.6%), consisting of* Ligusticum chuanxiong *Hort. And *Borneolum syntheticum. *
- Finally, three (4.6%) studies tested Xuezhikang capsule, which comprise partially purified extract of fermented *Monascus purpureus. *

### 3.3. Risk of Bias

**Figure 2:**
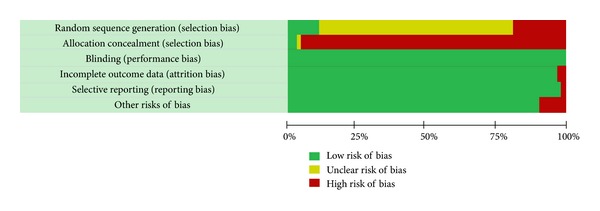
Risk of bias amongst included studies: mortality as primary outcome.

Among these 65 RCTs, only 7 were at low risk for bias for allocation sequence generation. Twelve were at high risk and the remaining RCTs did not report their sequence generation procedure clearly. All but one had high risk of bias in terms of allocation concealment and none of the included studies report the use of blinding. However, we regarded the risks of bias associated with lack of blinding and allocation concealment to be minimal, as the primary outcomes were of objective nature. Two of the included studies had high risk of bias for incomplete data and one for selective outcome reporting. Six are at high risk of bias due to other reasons. In summary, we consider the overall risk of bias amongst our included studies to be moderate (Figure 2). The detailed risk of bias assessment results is presented in Appendix 2 in the supplementary materials.

### 3.4. Effects of Interventions

#### 3.4.1. Impact on Fatal Outcomes

**Figure 3:**
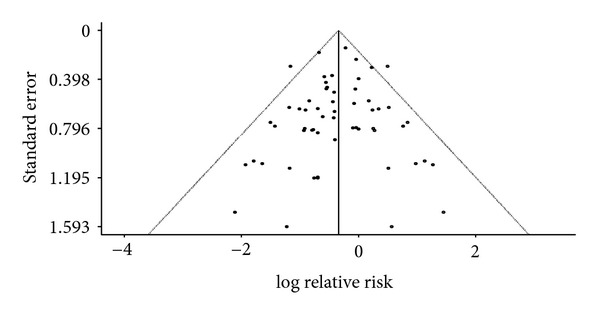
Trim and fill funnel plot on the prevention of all-cause mortality.

In this comparison (Table 2), a total of 44 RCTs reported total all-cause mortality. Pooled results demonstrated superiority of combined treatment in preventing all-cause mortality (RRR = 37%, 95% CI = 28%–45%). Funnel plot indicates the presence of publication bias. After applying trim and fill procedure (Figure 3), the RRR remained to be significant (RRR = 29%, 95% CI = 16%–40%, Table 2). Ten RCTs reported death of cardiac origin, and pooled findings also favor combined treatment (RRR = 39%, 95% CI = 22%–52%). Funnel plot indicates the presence of publication bias. After applying trim and fill procedure, the RRR remained to be significant (RRR = 32%, 95% CI = 15%–46%).

Pooled results from another four RCTs reporting the occurrence of fatal cardiogenic shock also favored combined treatment (RRR = 28%, 95% CI = 5%–45%). Respectively nine, six, five, and three RCTs reported outcomes on sudden cardiac death, fatal myocardial reinfarction, fatal heart failure, and fatal cardiac arrhythmia. In these four comparisons, all pooled findings favored combined treatment (sudden cardiac death: RRR = 24%, 95% CI = 6%–45%; fatal cardiac reinfarction: RRR = 54%, 95% CI = 12%–81%; fatal heart failure: RRR = 52%, 95% CI = 9%–79%; fatal cardiac arrhythmia: RRR = 29%, 95% CI = 84%–222%), but the estimates were statistically insignificant. Except for fatal myocardial reinfarction (*I* ^2^ = 37.3%), no significant heterogeneity existed in the comparisons mentioned above. However, given the small number of RCTs reporting this outcome, we were unable to explore heterogeneity using metaregression. 

#### 3.4.2. Impact on Nonfatal Cardiovascular Events

In this comparison (Table 2), a total of 11 RCTs reported overall, undifferentiated nonfatal heart events. Pooled results demonstrated superiority of combined treatment in preventing this outcome (RRR = 48%, 95% CI = 40%–56%). Twenty-three RCTs evaluated myocardial reinfarction, and the pooled result favors combined treatment (RRR = 52%, 95% CI = 39%–61%). The pooled results from 14 and 24 RCTs have also favored combined treatment, respectively, in preventing cardiogenic shock (RRR = 37%, 95% CI = 15%–53%) and in alleviating angina symptoms (RRR = 53%, 95% CI = 46%–61%). Three RCTs investigated nonfatal cardiac rupture as an outcome.The pooled finding supports combined treatment but the estimate was statistically insignificant (RRR = 56%, 95% CI = 67%–89%). No significant heterogeneity existed in all meta-analyses mentioned above.

Respectively, thirty and twenty-eight RCTs reported outcomes of cardiac arrhythmia and heart failure. In these two groups of studies, pooled findings all favored combined treatment, but high level of heterogeneity existed in both estimates (cardiac arrhythmia: RRR = 41%, 95% CI = 27%–52%, *I* ^2^ = 76.2%; heart failure: RRR = 48%, 95% CI = 36%–58%, *I* ^2^ = 47.9%). 

#### 3.4.3. Metaregression

We explored these heterogeneities by performing multivariate metaregression analyses using mean age, treatment duration, route of administration (oral versus intravenous), and baseline risk as covariates. None of the four covariates is significantly associated with cardiac arrhythmia (for baseline risk regression coefficient (*β*) = 0.46, *P* = 0.41; for mean age *β* = 0.00, *P* = 0.96; for duration of treatment *β* = 0.00, *P* = 0.95; for route of administration *β* = 0.21, *P* = 0.63), or heart failure (for baseline risk *β* = 0.67, *P* = 0.39; for mean age *β* = 0.02, *P* = 0.57; for duration of treatment *β* = 0.00, *P* = 0.87; for route of administration *β* = 0.12, *P* = 0.77). 

#### 3.4.4. CHM and BM versus BM Alone for MI: Adverse Events

In this comparison (Table 2), nine RCTs reported bleeding as adverse events, but the pooled estimate was statistically insignificant (RR = 0.97, 95% CI = 0.73, 1.28). Two RCTs reported general, undifferentiated adverse events, pooled estimate is heterogeneous and statistically insignificant (RR = 1.16, 95% CI = 0.59, 2.27, *I* ^2^ = 54.4%). 

## 4. Discussion 

This systematic review on the add-on effect of CHM on BM in the treatment of MI summarized findings from 12,022 patients reported in 65 RCTs. The overall risk of bias amongst included studies was moderate. Despite the lack of allocation concealment and blinding in the majority of included trials, its impact on risk of bias was less critical as we focused on objective outcomes. Random-effect meta-analyses demonstrated that combined treatment is superior to BM alone in reducing the risk of all-cause mortality and death of cardiac origin. Funnel plots indicated the presence of publication bias for both outcomes, and trim and fill procedures were conducted as sensitivity analyses. The directions of effect did not change after the adjustment, and the 95% CI of the estimates overlapped with the unadjusted values. The lower 95% CI boundary of the trim- and fill-adjusted RRR for all-cause and cardiac mortality was 16% and 15%, respectively. Conservatively speaking, CHM appeared to offer a protective add-on effect against mortality after adjusting for the publication bias, a common problem amongst the clinical research literature on CHM [87]. 

Combined treatment is also found to be more effective than BM alone in lowering the risk of fatal cardiogenic shock. Our analyses did not demonstrate therapeutic benefits of combined treatment on other reviewed fatal outcomes including myocardial reinfarction, cardiac arrhythmia, heart failure, and sudden cardiac death. For nonfatal outcomes, our analyses demonstrated that CHM is an effective add-on for lowering the risk of cardiogenic shock, cardiac arrhythmia, myocardial reinfarction, and the occurrence of total heart events. Benefits in preventing heart failure and angina were also observed but these findings are less robust given the subjective nature of the outcome, and metaregression did not shed light on potential sources of heterogeneity. We have considered including allocation concealment and blinding as covariates in our metaregressions but numbers of trials with low risk in these domains are too small for conducting such analysis. The effect of combined treatment on these two outcomes would need to be further evaluated with methodologically stronger trials. In addition, more comprehensive reporting on BM treatment details and adverse events is expected in future studies, preferably with reference to the CONSORT statement. 

Comprehensiveness of search is the major strength of this systematic review. The use of both international and Chinese databases allowed us to locate a much higher number studies compared to seven existing reviews on the topic [88]. We also attempted to synthesize results from trials evaluating heterogeneous CHM using random-effect model. This allowed us to estimate the average effect of adding CHM on top of conventional therapies [12]. The use of the trim and fill method has also partly circumvented the problem of publication bias. Nevertheless, the robustness of our conclusion depends on the assumption that the objective nature of outcomes was less affected by two major sources of bias: allocation concealment and blinding. While this assumption is tested in metaepidemiological studies [89, 90], the generalizability of these findings warrants further investigations.

## 5. Conclusion

Based on RCTs of moderate quality, this systematic review demonstrated consistent, add-on benefits of using CHM on top in BM treatment for preventing all-cause and cardiac mortality amongst MI patients. These findings are in line with the results from seven existing systematic reviews of smaller scope and lower methodological quality. This tentative conclusion warrants further scrutiny using rigorously designed RCT, and a more comprehensive approach in reporting BM treatment details and adverse events is warranted. 

## Supplementary Material

Search strategies and the detailed risk of bias assessment results are presented in appendices 1 and 2, respectively.

## Figures and Tables

**Table 1:** Main characteristic of included studies.

First author	Year	No. of patients in the treatment group	No. of patients in the control group	Diagnostic criteria	Intervention	Control	Duration of treatment (days)	Duration of followup (months)
CHD Group [21]	1981	138	138	Not reported	Kangxingeng heji + BM	BM	N/A	N/A
Kou [22]	1983	133	135	WHO criteria	Yiqihuoxue decoction and In + Xuejie powder + BM	BM	N/A	N/A
Chen [23]	1984	112	112	WHO criteria	Yiqihuoxue decoction + Yiqihuoxue In + BM	BM	35	N/A
Liang [24]	1989	74	74	Author defined	Tuoqingyanhu su + BM	BM	N/A	N/A
Xia [25]	1993	23	10	Not reported	Dushen tang + thrombolysis	Thrombolysis	3	N/A
Li [26]	1994	60	64	WHO criteria	Wenyanghuoxue decoction + BM	BM	14	0.1
Li [27]	1994	18	15	WHO criteria	Huangqi In + polarized solution	BM + polarized solution	28	1
Yang [28]	1997	66	80	WHO criteria	Shexiangbaoxin tablets + BM	BM	360	12
Zhang [29]	1998	76	59	WHO criteria	JianXin tablet + BM	BM	30	1
Guo [30]	1999	243	259	WHO criteria	Shenmai In + thrombolysis	Thrombolysis	14	1.25
Li [31]	1999	51	50	WHO criteria	Ligustrazine + compound danshen In + Chinese medicinal formulae + thrombolysis	Thrombolysis	28	1
Zhang [32]	1999	52	47	WHO criteria	Yiqihuoxuetongluo decoction + BM	BM	28	1
Guo [33]	2000	143	159	WHO criteria	Suxiao jiuxin pills + thrombolysis	Thrombolysis	14	1.25
Han [34]	2000	38	44	WHO criteria	Huangqi In + thrombolysis	Thrombolysis	10	1
Li [35]	2000	28	19	WHO criteria	Zhupi decoction + BM	BM	7	0.25
Li QZ (a) [36]*	2000	66	80	WHO criteria	Suxiao jiuxin pills + BM	BM	360	12
Li QZ (b) [36]*	2000	66	72	WHO criteria	Suxiao jiuxin pills + BM	BM + Propranolol	360	12
Lu [37]	2000	21	21	WHO criteria	Shuizhi In + BM	BM	14	0.5
Yin [38]	2000	15	13	WHO criteria	Shenmai In + Herba Erigerontis In + BM + thrombolysis	BM + Thrombolysis	14	N/A
Wu [39]	2001	54	49	WHO criteria	Huangqi In + Dan-Shen In + BM	BM	14	0.75
Bai [40]	2002	62	60	WHO criteria	Shenmai In + BM	BM	14	N/A
Shi [41]	2002	58	56	Author defined	Breviscapinun + BM + thrombolysis	BM + Thrombolysis	20	0.67
Guan [42]	2003	30	30	WHO criteria	Xingding In + BM	BM	15	1
Zhang [43]	2003	45	45	Not reported	Shenfu decoction+Xuefuzhupi decoction + BM	BM	28	N/A
Han [44]	2004	46	52	WHO criteria	Shexiangbaoxin tablets + BM + thrombolysis	BM + Thrombolysis	28	1
Li [45]	2004	32	18	WHO criteria	Shexiangbaoxin tablets + BM + thrombolysis	BM + Thrombolysis + placebo	90	3
Liu [46]	2004	41	96	WHO criteria	Shenmai In + BM	BM	15	N/A
Yang [47]	2004	45	45	Not reported	Huangqi In + thrombolysis	Thrombolysis	7	6
Chen [48]	2005	35	34	Not reported	Huangqi In + thrombolysis	Thrombolysis	10	12
Deng [49]	2005	38	35	Not reported	Xingnaojing In + BM	BM	21	24
He [50]	2005	23	23	Author defined	Kaixin capsule + BM + thrombolysis	BM + thrombolysis	5	0.17
Li [51]	2005	83	83	Not reported	Diaohuangqi In + BM	BM	28	2
Liu [52]*	2005	30	22	WHO criteria	Treatment based on TCM syndrome differentiation + thrombolysis	Thrombolysis	28	1
Miao [53]	2005	64	62	WHO criteria	Shengmai In + BM + thrombolysis	BM + thrombolysis	15	2
Yang [54]	2005	45	45	Criteria from the Chinese Society of Cardiology	Shexiangboxin tablets + BM	BM	N/A	3
Ding [55]	2006	15	15	WHO criteria	Shengmai In + BM	BM	N/A	N/A
Du (a)[56]*	2006	1364	1371	Not reported	Xuezhikang capsules + BM	BM + placebo	N/A	84
Du (b) [56]*	2006	1070	1065	Not reported	Xuezhikang capsules + BM	BM + placebo	1440	48
Li [57]	2006	31	32	Guideline from the European Society of Cardiology	Shenfu In + BM	BM	14	N/A
Ma [58]	2006	25	25	Criteria from the Chinese Society of Cardiology	Yuxingeng decoction + BM	BM	28	1
Qi [59]	2006	48	46	WHO criteria	Tanshinone II A sulfoacid In + BM + thrombolysis + PCI	BM + thrombolysis + PCI	14	0.5
Shen [60]	2006	83	82	Author defined	Shenfu In + BM	BM	N/A	0.25
Wang [61]	2006	228	162	WHO criteria	Shenmai In + BM	BM	14	1
Wei [62]	2006	31	37	WHO criteria	Shenfu In + BM + thrombolysis	BM + thrombolysis	7	0.25
Wu [63]	2006	19	21	WHO criteria	Shenmai In + BM	BM	20	1
Yang [64]	2006	48	49	Not reported	Xuezhikang capsules + BM	BM + placebo	N/A	72
Chen [65]	2007	30	30	WHO criteria	Tongxinluo capsule + BM	BM	56	2
Li [66]	2007	45	45	Author defined	Guanxinning In + BM	BM	15	6
Liang [67]	2007	90	68	WHO criteria	Shengmai In or Shenmai In + treatment based on TCM syndrome Differentiation + BM + thrombolysis	BM	N/A	N/A
Pan [68]	2007	20	20	WHO criteria	Tongxinluo capsule + BM	BM	N/A	1
Zhai [69]	2007	38	30	Criteria from Chinese Society of Cardiology	Shenmai In + BM	BM	10	N/A
Ding [70]	2008	23	23	Author defined	Shengmai In + BM	BM	N/A	N/A
Lan [71]	2008	130	128	WHO criteria	Xinmaitong capsules + BM	BM	30	1
Yu [72]	2008	100	96	Author defined	Shexiang Baoxin tablets + BM	BM	10	1
Yu [73]	2008	32	32	Author defined	Yinxingdamo In + BM + thrombolysis	BM + thrombolysis	14	0.5
Zhang [74]	2008	27	27	WHO criteria	Shenmai In + Shuxuening In + BM	BM	28	N/A
Gao [75]	2009	60	60	WHO criteria	Danhong In + BM	BM	N/A	0.5
Lin [76]	2009	25	25	Author defined	Compound danshen dripping pills + BM	BM	N/A	1
Liu [77]	2009	16	16	Author defined	Tanshinone II A sulfoacid In + BM	BM	7	3
Song [78]	2009	36	34	Author defined	Tongxinluo capsule + Shenmai In + Gegensu In + BM	BM	28	N/A
Yuan [79]	2009	38	38	WHO criteria	Shenmai In + thrombolysis	Thrombolysis	10	1
Zhao [80]	2009	50	48	Not reported	Tongxinluo capsule + BM + thrombolysis	BM + thrombolysis	N/A	12
Zuo [81]	2009	80	80	Criteria from Chinese Society of Cardiology	Breviscapinun + BM	BM	14	1
Guo [82]	2010	48	45	Not reported	Compound Danshen tablet + BM	BM	N/A	12
Xu [83]	2010	32	30	Author defined	Treatment based on TCM syndrome differentiation + BM	BM	28	1

BM: routine biomedical treatment as defined by the investigators; In: injection; N/A: not reported.

*Two RCTs reported in one publication.

**Table 2:** Chinese herbal medicine plus biomedical treatment versus biomedical treatment alone for treating myocardial infarction: random-effect meta-analysis.

Events	No. of studies	No. of events/total no.		Combined effect			Test for heterogeneity			Adjusted combined effect (trim and fill)		
		CHM + BM group	BM group	RR (95% CI)	RRR (%) (95% CI)	*P* value^#^	*χ* ^2^ statistic	*P* value*	*I* ^2^ (%)	RR (95% CI)	RRR (95% CI)	*P* value^#^
Fatal events												
All-cause mortality	44	308/5107	521/5112	0.63 (0.55–0.72)	37% (28%–45%)	<0.001	37.47	0.709	0.0	0.71 (0.60–0.84)	29% (16%–40%)	<0.001
Mortality of cardiac origin	10	142/2820	227/2796	0.61 (0.48–0.78)	39% (22%–52%)	<0.001	11.66	0.233	22.8	0.68 (0.54–0.85)	32% (15%–46%)	0.001
Fatal myocardial reinfarction	6	20/2660	37/2687	0.46 (0.19–1.12)	54% (12%–81%)	0.086	7.98	0.157	37.3	—		—
Fatal cardiac arrhythmia	3	4/162	5/160	0.71 (0.16–3.22)	29% (84%–222%)	0.662	2.21	0.331	9.6	—		—
Fatal heart failure	5	8/410	18/444	0.48 (0.21–1.09)	52% (9%–79%)	0.078	0.06	1.000	0.0	—		—
Fatal cardiogenic shock	4	37/330	58/332	0.72 (0.55–0.95)	28% (5%–45%)	0.019	2.42	0.490	0.0	—		—
Sudden cardiac death	9	61/2775	81/2795	0.76 (0.55–1.06)	24% (6%–45%)	0.104	3.13	0.926	0.0	—		—
Nonfatal events												
Undifferentiated total heart events	11	209/2762	407/2761	0.52 (0.44–0.60)	48% (40%–56%)	<0.001	8.99	0.533	0.0	0.52 (0.44–0.62)	48% (38%–56%)	<0.001
Myocardial reinfarction	23	103/2377	215/2343	0.48 (0.39–0.61)	52% (39%–61%)	<0.001	9.95	0.987	0.0	0.53 (0.43–0.66)	47% (34%–57%)	<0.001
Cardiac arrhythmia	30	398/1730	640/1696	0.59 (0.48–0.73)	41% (27%–52%)	<0.001	121.94	0.000	76.2	0.72 (0.58–0.89)	28% (11%–42%)	0.003
Heart failure	28	249/1825	496/1835	0.52 (0.42–0.64)	48% (36%–58%)	<0.001	51.86	0.003	47.9	0.60 (0.50–0.72)	40% (28%–50%)	<0.001
Cardiac rupture	3	2/122	7/134	0.44 (0.11–1.67)	56% (67%–89%)	0.224	0.60	0.740	0.0	—		—
Cardiogenic shock	14	63/1015	110/1030	0.63 (0.47–0.85)	37% (15%–53%)	0.002	10.65	0.640	0.0	0.75 (0.57–0.98)	25% (2%–43%)	0.036
Angina	24	177/1047	297/1001	0.47 (0.39–0.56)	53% (44%–61%)	<0.001	22.20	0.508	0.0	0.58 (0.48–0.69)	42% (31%–52%)	<0.001
Adverse events												
Undifferentiated total events	2	43/2434	39/2436	1.16 (0.59–2.27)	16% (41%–127%)	0.664	2.19	0.138	54.4	—		—
Bleeding	9	81/706	81/745	0.97 (0.73–1.28)	3% (27%-28%)	0.816	4.89	0.769	0.0	—		—

^#^Test for overall effect; *chi-square test for heterogeneity.

BM: biomedical treatment; CHM: Chinese herbal medicine treatment; RR: relative risk; RRR: relative risk reduction; 95% CI: 95% confidence interval.
