# Supplementary material for: Add-On Effect of Chinese Herbal Medicine on Mortality in Myocardial Infarction: Systematic Review and Meta-Analysis of Randomized Controlled Trials
Source: Evid Based Complement Alternat Med. 2013 Jan 10;2013:675906. doi: 10.1155/2013/675906 (PMC3556418; doi:10.1155/2013/675906)
Supplement: Supplementary file 1 — Search strategies and the detailed risk of bias assessment results are presented in appendices 1 and 2, respectively. [file 675906.f1.doc]

**Appendix 1: Search strategies and results**

MEDLINE

Search time: 28 June 2010

1 Clinical trial.mp. (513559)

2 clinical trial.pt. (462927)

3 random:.mp. (637094)

4 tu.xs. (2916488)

5 randomized controlled trial.pt. (293863)

6 controlled clinical trial.pt. (81845)

7 placebo.ab. (120044)

8 drug therapy.fs. (1391575)

9 trial.ab. (207828)

10 trial.mp. (672924)

11 trial$.mp. (908556)

12 groups.ab. (978640)

13 group$.mp. (2004834)

14 1 or 2 or 3 or 4 or 5 or 6 or 7 or 8 or 9 or 10 or 11 or 12 or 13 (5019226)

15 exp Myocardial Ischemia/ (309698)

16 myocard$ ischemi$.mp. (40327)

17 exp Heart Diseases/ (754872)

18 heart disease$.mp. (151767)

19 angina.mp. (53904)

20 (heart adj3 disease$).mp. (167534)

21 (coronary adj3 disease$).mp. (178631)

22 myocardial infarct$.mp. (158996)

23 exp Myocardial Revascularization/ (67471)

24 myocardi$ revascular$.mp. (10317)

25 (coronary adj3 bypass$).mp. (46614)

26 cabg.mp. (9383)

27 (coronary adj3 angioplast$).mp. (31563)

28 ptca.mp. (6025)

29 (heart adj3 infarct$).mp. (5615)

30 postmyocardial infarct$.mp. (730)

31 15 or 16 or 17 or 18 or 19 or 20 or 21 or 22 or 23 or 24 or 25 or 26 or 27 or 28 or 29 or 30 (826923)

32 exp Drugs, Chinese Herbal/ (19792)

33 Chinese herb$.mp. (20924)

34 exp Medicine, Chinese traditional/ (8556)

35 Traditional Chinese medic$.mp. (4943)

36 exp Phytotherapy/ (21333)

37 phytother$.mp. (21442)

38 (chinese adj5 (traditional or medicine$)).mp. (14635)

39 (herbs or herbal).mp. (30662)

40 (plant or plants).mp. (364139)

41 (traditional adj5 medicine$).mp. (23836)

42 (She Xiang Bao Xin or Su Xiao Jiu Xin or Fu Fang Dan Shen or Guan Xin Dan Shen or Su Bing or Su Xin or Tong Xin Luo or Hu Xin or Jiu Xin or Huan Xin or She Xiang Su He or She Xiang or Zhu She Xiao Shuan or Huo Xin Dan or Yi Xin Wan or Xin Ling Wan or She Xiang Xin Nao Le or Kuan Xiong or Xin Tong or Fu Fang Xi Xin or Guan Xin).mp. (22)

43 (Xin Jiao Tong Ning or Jiu Xin or Ge Gen Su or Shen Fu or Di Long or Hong Hua or Shu Xue Ning or Chuan Qiong Chin or Shu Xue Tong or Mai Pu Lin or Deng Zhan Xi Xin or Huang Qi or Ci Wu Jia or Guan Xin Ning or Tong Xin Luo or Nao Xin Tong or Bu Xin qi or Shen Shao or Nuo Di Kang or Yang Xin Shi or Xue Fu Zhu Yu or Xin Ke Shu).mp. (110)

44 (Di Ao Xin Xue Kang or Xin Yuan or Wen Xin or Sheng Mai or Yi Xin Shu or Xin Nao Shu Tong or Yin Xing Ye or Huo Xue Tong Mai or Xin Ke Ning or Xue Sai Tong or Xin Tong or Dan Lou or Xin Bao or You Gui or Xin Mai Tong or Tong Guan or Shen Mai or Deng Zhan Hua).mp. (51)

45 32 or 33 or 34 or 35 or 36 or 37 or 38 or 39 or 40 or 41 or 42 or 43 or 44 (399403)

46 14 and 31 and 45 (3609)

47 limit 46 to humans (2680)

48 limit 47 to ("article reviews (acp journal club)" or "article reviews (dare)" or evidence based medicine reviews or "topic reviews (cochrane)") (46)

49 limit 47 to "review" (579)

50 48 or 49 (603)

51 47 not 50 (2077)

1. from 51 keep 1-2077 (2077)

CENTRAL:

Search time: 28 June 2010

1 exp Myocardial Ischemia/ (16340)

2 exp Heart Diseases/ (26917)

3 heart disease$.mp. (6541)

4 Myocardi$ Ischem$.mp. (2379)

5 exp Myocardial Revascularization/ (6464)

6 Myocardi$ Revascular$.mp. (817)

7 angina.mp. (6875)

8 (heart adj3 disease$).mp. (7160)

9 (coronary adj3 disease).mp. (10808)

10 (myocardial adj1 infarct$).mp. (11216)

11 (coronary adj3 bypass$).mp. (5512)

12 exp Coronary Artery Bypass/ (3887)

13 (coronary adj3 angioplast$).mp. (3186)

14 exp Angioplasty, Transluminal, Percutaneous Coronary/ (2515)

15 (heart adj3 infarct$).mp. (1429)

16 postmyocardial.mp. (90)

17 1 or 2 or 3 or 4 or 5 or 6 or 7 or 8 or 9 or 10 or 11 or 12 or 13 or 14 or 15 or 16 (38693)

18 exp Drugs, Chinese Herbal/ (1664)

19 chinese herb$.mp. (1943)

20 exp Medicine, Chinese Traditional/ (468)

21 traditional chinese med$.mp. (605)

22 exp Medicine, East Asian Traditional/ (508)

23 (chinese adj (traditional or medicine$)).mp. (1363)

24 (herbs or herbal).mp. (2721)

25 (plant or plants).mp. (4837)

26 exp Phytotherapy/ (2172)

27 (traditional adj3 medicine$).mp. (1439)

28 (She Xiang Bao Xin or Su Xiao Jiu Xin or Fu Fang Dan Shen or Guan Xin Dan Shen or Su Bing or Su Xin or Tong Xin Luo or Hu Xin or Jiu Xin or Huan Xin or She Xiang Su He or She Xiang or Zhu She Xiao Shuan or Huo Xin Dan or Yi Xin Wan or Xin Ling Wan or She Xiang Xin Nao Le or Kuan Xiong or Xin Tong or Fu Fang Xi Xin or Guan Xin).mp. (22)

29 (Xin Jiao Tong Ning or Jiu Xin or Ge Gen Su or Shen Fu or Di Long or Hong Hua or Shu Xue Ning or Chuan Qiong Chin or Shu Xue Tong or Mai Pu Lin or Deng Zhan Xi Xin or Huang Qi or Ci Wu Jia or Guan Xin Ning or Tong Xin Luo or Nao Xin Tong or Bu Xin qi or Shen Shao or Nuo Di Kang or Yang Xin Shi or Xue Fu Zhu Yu or Xin Ke Shu).mp. (46)

30 (Di Ao Xin Xue Kang or Xin Yuan or Wen Xin or Sheng Mai or Yi Xin Shu or Xin Nao Shu Tong or Yin Xing Ye or Huo Xue Tong Mai or Xin Ke Ning or Xue Sai Tong or Xin Tong or Dan Lou or Xin Bao or You Gui or Xin Mai Tong or Tong Guan or Shen Mai or Deng Zhan Hua).mp. (39)

31 18 or 19 or 20 or 21 or 22 or 23 or 24 or 25 or 26 or 27 or 28 or 29 or 30 (8619)

32 17 and 31 (479)

EMABSE

Search time: 28 June 2010

1 random$.mp. (493021)

2 factorial$.mp. (18460)

3 (crossover$ or cross-over$).mp. (47919)

4 placebo$.mp. (198663)

5 (doubl$ adj blind$).mp. (114351)

6 (singl$ adj blind$).mp. (14041)

7 assign$.mp. (122600)

8 allocat$.mp. (45111)

9 volunteer$.mp. (112947)

10 Crossover Procedure.sh. (23240)

11 Double-blind Procedure.sh. (78562)

12 Randomized Controlled Trial.sh. (191367)

13 Single-blind Procedure.sh. (9619)

14 random:.tw. (446443)

15 clinical trial:.mp. (677850)

16 exp health care quality/ (926899)

17 (random$ or placebo$).ti,ab. (497960)

18 ((single$ or double$ or triple$ or treble$) and (blind$ or mask$)).ti,ab. (108350)

19 controlled clinical trial$.ti,ab. (11693)

20 RETRACTED ARTICLE/ (3431)

21 Clinical trial/ (605247)

22 Randomized controlled trial/ (191367)

23 Randomization/ (28492)

24 Single blind procedure/ (9619)

25 Double blind procedure/ (78562)

26 Crossover procedure/ (23240)

27 Placebo/ (144300)

28 Randomi?ed controlled trial$.tw. (41070)

29 Rct.tw. (3639)

30 Random allocation.tw. (700)

31 Randomly allocated.tw. (11275)

32 Allocated randomly.tw. (1420)

33 (allocated adj2 random).tw. (575)

34 Single blind$.tw. (8228)

35 Double blind$.tw. (91015)

36 (triple adj blind$).tw. (153)

37 Placebo$.tw. (120147)

38 Prospective study/ (97004)

39 1 or 2 or 3 or 4 or 5 or 6 or 7 or 8 or 9 or 10 or 11 or 12 or 13 or 14 or 15 or 16 or 17 or 18 or 19 or 20 or 21 or 22 or 23 or 24 or 25 or 26 or 27 or 28 or 29 or 30 or 31 or 32 or 33 or 34 or 35 or 36 or 37 or 38 (1928006)

40 Case study/ (7285)

41 Case report.tw. (133625)

42 Abstract report/ or letter/ (543440)

43 40 or 41 or 42 (681573)

44 39 not 43 (1825352)

45 exp Ischemic heart disease/ (256257)

46 exp Heart Diseases/ (656662)

47 angina.mp. (48943)

48 (heart adj3 disease$).mp. (149300)

49 (coronary adj3 disease$).mp. (106491)

50 myocardial infarct$.mp. (93824)

51 exp Myocardial Revascularization/ (13215)

52 (coronary adj3 bypass$).mp. (43232)

53 cabg.mp. (8680)

54 (coronary adj3 angioplast$).mp. (21264)

55 ptca.mp. (5670)

56 heart infarct$.mp. (132549)

57 postmyocardial infarct$.mp. (616)

58 45 or 46 or 47 or 48 or 49 or 50 or 51 or 52 or 53 or 54 or 55 or 56 or 57 (728538)

59 exp Chinese medicine/ (10161)

60 exp oriental medicine/ (136)

61 exp herbaceous agent/ (13268)

62 exp medicinal plant/ (55133)

63 chinese medicine.mp. (12463)

64 chinese traditional medicine.mp. (383)

65 traditional chinese medicine.mp. (4641)

66 oriental medicine.mp. (470)

67 traditional oriental medicine.mp. (89)

68 oriental traditional medicine.mp. (21)

69 chinese herb$.mp. (4719)

70 chinese medicinal.mp. (1055)

71 medicinal plant$.mp. (23071)

72 herb$.mp. (50176)

73 (She Xiang Bao Xin or Su Xiao Jiu Xin or Fu Fang Dan Shen or Guan Xin Dan Shen or Su Bing or Su Xin or Tong Xin Luo or Hu Xin or Jiu Xin or Huan Xin or She Xiang Su He or She Xiang or Zhu She Xiao Shuan or Huo Xin Dan or Yi Xin Wan or Xin Ling Wan or She Xiang Xin Nao Le or Kuan Xiong or Xin Tong or Fu Fang Xi Xin or Guan Xin).mp. (23)

74 (Xin Jiao Tong Ning or Jiu Xin or Ge Gen Su or Shen Fu or Di Long or Hong Hua or Shu Xue Ning or Chuan Qiong Chin or Shu Xue Tong or Mai Pu Lin or Deng Zhan Xi Xin or Huang Qi or Ci Wu Jia or Guan Xin Ning or Tong Xin Luo or Nao Xin Tong or Bu Xin qi or Shen Shao or Nuo Di Kang or Yang Xin Shi or Xue Fu Zhu Yu or Xin Ke Shu).mp. (97)

75 (Di Ao Xin Xue Kang or Xin Yuan or Wen Xin or Sheng Mai or Yi Xin Shu or Xin Nao Shu Tong or Yin Xing Ye or Huo Xue Tong Mai or Xin Ke Ning or Xue Sai Tong or Xin Tong or Dan Lou or Xin Bao or You Gui or Xin Mai Tong or Tong Guan or Shen Mai or Deng Zhan Hua).mp. (50)

76 59 or 60 or 61 or 62 or 63 or 64 or 65 or 66 or 67 or 68 or 69 or 70 or 71 or 72 or 73 or 74 or 75 (99567)

77 39 and 58 and 76 (1487)

78 limit 77 to human (1321)

79 limit 78 to "review" (595)

80 78 not 79 (726)

AMED

Search time: 28 June 2010

1 Controlled study/ or Randomized Controlled Trial/ (1381)

2 Placebo/ (506)

3 Random$.tw. (11231)

4 latin square.tw. (24)

5 crossover.tw. (514)

6 cross-over.tw. (219)

7 placebo$.tw. (2345)

8 ((doubl$ or singl$ or tripl$ or trebl$) adj5 (blind$ or mask$)).tw. (1995)

9 (comparativ$ adj5 trial$).tw. (139)

10 (clinical adj5 trial$).tw. (4166)

11 exp traditional medicine chinese/ (4744)

12 exp drugs chinese herbal/ (1348)

13 exp plants medicinal/ (16139)

14 exp herbal drugs/ (6618)

15 herb$.mp. (10453)

16 (chinese adj5 medicine).mp. (5058)

17 (traditional chinese adj5 medicine).mp. (878)

18 (chinese adj5 drug$).mp. (1538)

19 (chinese adj5 herb$).mp. (1780)

20 (medic$ adj5 plant$).mp. (15307)

21 (herb$ adj5 plant$).mp. (442)

22 (She Xiang Bao Xin or Su Xiao Jiu Xin or Fu Fang Dan Shen or Guan Xin Dan Shen or Su Bing or Su Xin or Tong Xin Luo or Hu Xin or Jiu Xin or Huan Xin or She Xiang Su He or She Xiang or Zhu She Xiao Shuan or Huo Xin Dan or Yi Xin Wan or Xin Ling Wan or She Xiang Xin Nao Le or Kuan Xiong or Xin Tong or Fu Fang Xi Xin or Guan Xin).mp. (3)

23 (Xin Jiao Tong Ning or Jiu Xin or Ge Gen Su or Shen Fu or Di Long or Hong Hua or Shu Xue Ning or Chuan Qiong Chin or Shu Xue Tong or Mai Pu Lin or Deng Zhan Xi Xin or Huang Qi or Ci Wu Jia or Guan Xin Ning or Tong Xin Luo or Nao Xin Tong or Bu Xin qi or Shen Shao or Nuo Di Kang or Yang Xin Shi or Xue Fu Zhu Yu or Xin Ke Shu).mp. (11)

24 (Di Ao Xin Xue Kang or Xin Yuan or Wen Xin or Sheng Mai or Yi Xin Shu or Xin Nao Shu Tong or Yin Xing Ye or Huo Xue Tong Mai or Xin Ke Ning or Xue Sai Tong or Xin Tong or Dan Lou or Xin Bao or You Gui or Xin Mai Tong or Tong Guan or Shen Mai or Deng Zhan Hua).mp. (9)

25 (coronary adj3 disease$).mp. (922)

26 angina.mp. (241)

27 myocardial infarct$.mp. (586)

28 heart attack$.mp. (52)

29 (cardiovascular adj2 (disease$ or event$)).mp. (1300)

30 exp cardiovascular disease/ (11782)

31 25 or 26 or 27 or 28 or 29 or 30 (12285)

32 11 or 12 or 13 or 14 or 15 or 16 or 17 or 18 or 19 or 20 or 21 or 22 or 23 or 24 (26067)

33 1 or 2 or 3 or 4 or 5 or 6 or 7 or 8 or 9 or 10 or 11 or 12 or 13 or 14 or 15 or 16 or 17 or 18 or 19 or 20 or 21 or 22 or 23 or 24 (39508)

34 31 and 32 and 33 (946)

35 limit 34 to "review" (11)

36 34 not 35 (935)

CINAHL Plus

Search time: 28 June 2010

**Records in total: 1618**

| S30 | S27 and S28 | Limiters - Clinical Queries: Therapy - High Sensitivity; Human  Search modes - Boolean/Phrase | Interface - EBSCOhost  Search Screen - Advanced Search  Database - CINAHL Plus |
| --- | --- | --- | --- |
| S29 | S27 and S28 | Search modes - Boolean/Phrase | Interface - EBSCOhost  Search Screen - Advanced Search  Database - CINAHL Plus |
| S28 | S14 or S15 or S16 or S17 or S18 or S19 or S20 or S21 or S22 or S23 or S24 or S25 | Search modes - Boolean/Phrase | Interface - EBSCOhost  Search Screen - Advanced Search  Database - CINAHL Plus |
| S27 | S13 or S26 | Search modes - Boolean/Phrase | Interface - EBSCOhost  Search Screen - Advanced Search  Database - CINAHL Plus |
| S26 | ("cardiovascular") or (MH "Cardiovascular Abnormalities+") or (MH "Cardiovascular Diseases+") or (MH "Cardiovascular System+") or (MH "Surgery, Cardiovascular+") or (MH "Technology, Cardiovascular") or (MH "Cardiovascular Care") or (MH "Cardiovascular Nursing+") | Search modes - Boolean/Phrase | Interface - EBSCOhost  Search Screen - Advanced Search  Database - CINAHL Plus |
| S25 | plant extract$ | Search modes - Boolean/Phrase | Interface - EBSCOhost  Search Screen - Advanced Search  Database - CINAHL Plus |
| S24 | Complementary Therap$ | Search modes - Boolean/Phrase | Interface - EBSCOhost  Search Screen - Advanced Search  Database - CINAHL Plus |
| S23 | alternative medicine | Search modes - Boolean/Phrase | Interface - EBSCOhost  Search Screen - Advanced Search  Database - CINAHL Plus |
| S22 | (MH "Medicine, Chinese Traditional+") or (MH "Medicine, Oriental Traditional+") or (MH "Medicine, Traditional+") or (MH "Traditional Healers") | Search modes - Boolean/Phrase | Interface - EBSCOhost  Search Screen - Advanced Search  Database - CINAHL Plus |
| S21 | herb | Search modes - Boolean/Phrase | Interface - EBSCOhost  Search Screen - Advanced Search  Database - CINAHL Plus |
| S20 | Medicinal Plant/ | Search modes - Boolean/Phrase | Interface - EBSCOhost  Search Screen - Advanced Search  Database - CINAHL Plus |
| S19 | Chinese Herb/ | Search modes - Boolean/Phrase | Interface - EBSCOhost  Search Screen - Advanced Search  Database - CINAHL Plus |
| S18 | Chinese Drug/ | Search modes - Boolean/Phrase | Interface - EBSCOhost  Search Screen - Advanced Search  Database - CINAHL Plus |
| S17 | Plant Extract/ | Search modes - Boolean/Phrase | Interface - EBSCOhost  Search Screen - Advanced Search  Database - CINAHL Plus |
| S16 | chinese medicine/ | Search modes - Boolean/Phrase | Interface - EBSCOhost  Search Screen - Advanced Search  Database - CINAHL Plus |
| S15 | traditional medicine | Search modes - Boolean/Phrase | Interface - EBSCOhost  Search Screen - Advanced Search  Database - CINAHL Plus |
| S14 | alternative medicine/ | Search modes - Boolean/Phrase | Interface - EBSCOhost  Search Screen - Advanced Search  Database - CINAHL Plus |
| S13 | S1 or S2 or S3 or S4 or S5 or S6 or S7 or S8 or S9 or S10 or S11 or S12 | Search modes - Boolean/Phrase | Interface - EBSCOhost  Search Screen - Advanced Search  Database - CINAHL Plus |
| S12 | (coronary n3 bypass$) | Search modes - Boolean/Phrase | Interface - EBSCOhost  Search Screen - Advanced Search  Database - CINAHL Plus |
| S11 | (heart n3 disease$) | Search modes - Boolean/Phrase | Interface - EBSCOhost  Search Screen - Advanced Search  Database - CINAHL Plus |
| S10 | (myocardial n3 infarct$) | Search modes - Boolean/Phrase | Interface - EBSCOhost  Search Screen - Advanced Search  Database - CINAHL Plus |
| S9 | (heart n3 infarct$) | Search modes - Boolean/Phrase | Interface - EBSCOhost  Search Screen - Advanced Search  Database - CINAHL Plus |
| S8 | angina | Search modes - Boolean/Phrase | Interface - EBSCOhost  Search Screen - Advanced Search  Database - CINAHL Plus |
| S7 | (coronary n3 disease$) | Search modes - Boolean/Phrase | Interface - EBSCOhost  Search Screen - Advanced Search  Database - CINAHL Plus |
| S6 | MH heart function test | Search modes - SmartText Searching | Interface - EBSCOhost  Search Screen - Advanced Search  Database - CINAHL Plus |
| S5 | Heart Disease/ | Search modes - Boolean/Phrase | Interface - EBSCOhost  Search Screen - Advanced Search  Database - CINAHL Plus |
| S4 | MH Ischemic Heart Disease | Search modes - SmartText Searching | Interface - EBSCOhost  Search Screen - Advanced Search  Database - CINAHL Plus |
| S3 | Heart Disease | Search modes - Boolean/Phrase | Interface - EBSCOhost  Search Screen - Advanced Search  Database - CINAHL Plus |
| S2 | MH heart surgery+ | Search modes - Boolean/Phrase | Interface - EBSCOhost  Search Screen - Advanced Search  Database - CINAHL Plus |
| S1 | Cardiovascular Disease/ | Search modes - Boolean/Phrase | Interface - EBSCOhost  Search Screen - Advanced Search  Database - CINAHL Plus |

CBM (China Biological Medicine Database, 中国生物医学文献数据库)

Search time: 22 July 2010

|  | **Results** |  | **Search Strategy** |
| --- | --- | --- | --- |
| 1 | 110837 |  | 主题词:临床试验/全部树/全部副主题词 |
| 2 | 29819 |  | 主题词:随机分配/全部树/全部副主题词 |
| 3 | 3683 |  | 主题词:双盲法/全部树/全部副主题词 |
| 4 | 413 |  | 主题词:单盲法/全部树/全部副主题词 |
| 5 | 2599 |  | 主题词:安慰剂/全部树/全部副主题词 |
| 6 | 143021 |  | #5 or #4 or #3 or #2 or #1 |
| 7 | 2084 |  | 主题词=治疗应用 |
| 8 | 145039 |  | #7 or #6 |
| 9 | 457344 |  | 中文标题:随机对照 or 临床试验 or 临床研究 or 临床观察 or 临床效果 or 临床分析 or 临床疗效 or 临床比较 or 对照研究 or 对照试验 or 对照治疗 or 对照观察 or 对照分析 or 对比研究 or 对比观察 or 对比分析 or 分组研究　or 比较研究 or 多中心研究 or 疗效观察　or 疗效评价 or 疗效分析 or 疗效比较 or 治疗研究 or 治疗比较 or 效果比较 |
| 10 | 559316 |  | #9 or #8 |
| 11 | 7128 |  | 主题词:心肌缺血/全部树/ZD/ZJ |
| 12 | 54223 |  | 主题词:中草药△/全部树/AE/PO/TU |
| 13 | 67281 |  | 中文标题:丹参 or 葛根 or 细辛 or 红花 or 三七 or 刺五加 or 毛冬青 or 益母草 or 延胡索 or 赤芍 or 蒲黄 or 淫羊藿 or 瓜蒌 or 防己 or 灵芝 or 山楂 or 当归 or 川穹 or 麝香 or 苏合香 or 灯盏花 or 银杏叶 or 阿槐酸钠 or 速效救心 or 冠心苏合 or 苏心丸 or 益心丸 or 心灵丸 or 心宝 or 右归 or 护心丹 or 救心丹 or 环心丹 or 活心丹 or 苏冰滴丸 or 冠心膏 or 心绞痛宁膏 or 救心油 or 养心氏片 or 心可舒 or 稳心颗粒 or 丹七片 or 丹蒌片 or 心血宁 or 通心络 or 心脉通 or 脑心通 or 益心舒 or 心可宁 or 诺迪康 or 血脉康 or 心血康 or 珠麝消栓 or 心脑舒通 or 血府逐瘀 or 心元胶囊 or 通冠胶囊 or 活血通脉胶囊 or 补心气口服液 or 心通口服液 or 舒心口服液 or 滋心阴口服液 or 参芍 or 生脉 or 血塞通 or 血栓通 or 脉络宁 or 舒血宁 or 疏血通 or 冠心宁 or 脉普林 or 醒脑静 or 清开灵 or 参附注射液 or 参麦注射液 or 地龙注射液 or 黄芪注射液 or 宽胸气雾剂 or 心痛气雾剂 |
| 14 | 42506 |  | 关键词:丹参 or 葛根 or 细辛 or 红花 or 三七 or 刺五加 or 毛冬青 or 益母草 or 延胡索 or 赤芍 or 蒲黄 or 淫羊藿 or 瓜蒌 or 防己 or 灵芝 or 山楂 or 当归 or 川穹 or 麝香 or 苏合香 or 灯盏花 or 银杏叶 or 阿槐酸钠 or 速效救心 or 冠心苏合 or 苏心丸 or 益心丸 or 心灵丸 or 心宝 or 右归 or 护心丹 or 救心丹 or 环心丹 or 活心丹 or 苏冰滴丸 or 冠心膏 or 心绞痛宁膏 or 救心油 or 养心氏片 or 心可舒 or 稳心颗粒 or 丹七片 or 丹蒌片 or 心血宁 or 通心络 or 心脉通 or 脑心通 or 益心舒 or 心可宁 or 诺迪康 or 血脉康 or 心血康 or 珠麝消栓 or 心脑舒通 or 血府逐瘀 or 心元胶囊 or 通冠胶囊 or 活血通脉胶囊 or 补心气口服液 or 心通口服液 or 舒心口服液 or 滋心阴口服液 or 参芍 or 生脉 or 血塞通 or 血栓通 or 脉络宁 or 舒血宁 or 疏血通 or 冠心宁 or 脉普林 or 醒脑静 or 清开灵 or 参附注射液 or 参麦注射液 or 地龙注射液 or 黄芪注射液 or 宽胸气雾剂 or 心痛气雾剂 |
| 15 | 67857 |  | 主题词:丹参 or 葛根 or 细辛 or 红花 or 三七 or 刺五加 or 毛冬青 or 益母草 or 延胡索 or 赤芍 or 蒲黄 or 淫羊藿 or 瓜蒌 or 防己 or 灵芝 or 山楂 or 当归 or 川穹 or 麝香 or 苏合香 or 灯盏花 or 银杏叶 or 阿槐酸钠 or 速效救心 or 冠心苏合 or 苏心丸 or 益心丸 or 心灵丸 or 心宝 or 右归 or 护心丹 or 救心丹 or 环心丹 or 活心丹 or 苏冰滴丸 or 冠心膏 or 心绞痛宁膏 or 救心油 or 养心氏片 or 心可舒 or 稳心颗粒 or 丹七片 or 丹蒌片 or 心血宁 or 通心络 or 心脉通 or 脑心通 or 益心舒 or 心可宁 or 诺迪康 or 血脉康 or 心血康 or 珠麝消栓 or 心脑舒通 or 血府逐瘀 or 心元胶囊 or 通冠胶囊 or 活血通脉胶囊 or 补心气口服液 or 心通口服液 or 舒心口服液 or 滋心阴口服液 or 参芍 or 生脉 or 血塞通 or 血栓通 or 脉络宁 or 舒血宁 or 疏血通 or 冠心宁 or 脉普林 or 醒脑静 or 清开灵 or 参附注射液 or 参麦注射液 or 地龙注射液 or 黄芪注射液 or 宽胸气雾剂 or 心痛气雾剂 |
| 16 | 87956 |  | #13 or #14 or #15 |
| 17 | 128615 |  | #16 or #12 |
| 18 | 134169 |  | 主题词:心肌缺血/全部树/全部副主题词 |
| 19 | 9745 |  | #17 and #18 |
| 20 | 13049 |  | #19 or #11 |
| 21 | 4772 |  | #20 and #10 |
| 22 | 4523 |  | #20 and #10 -限定:人类 |
| 23 | 29 |  | #20 and #10 -限定:综述 |
| 24 | 0 |  | #20 and #10 -限定:讲座 |
| 25 | 0 |  | #20 and #10 -限定:译文 |
| 26 | 5 |  | #20 and #10 -限定:病例报告 |
| 27 | 13 |  | #20 and #10 -限定:Meta分析 |
| 28 | 47 |  | #27 or #26 or #25 or #24 or #23 |
| 29 | 4482 |  | (#22) not (#28) |
| 30 | 335093 |  | 中文标题:浅谈 or 浅析 or 初探 or 新用 or 巧用 or 漫谈 or 治验 or 体会 or 举验 or 举隅 or 举要 or 案例 or 刍议 or 经验 or 验案 or 配伍分析 or 概述 |
| 31 | 4473 |  | (#29) not (#30) |
| 32 | 13 |  | (#29) not (#30) -限定:动物 |
| 33 | 4460 |  | (#31) not (#32) |

CMCC ([Chinese Medical Current Contents](http://158.132.160.18/databases/cmcc), 中文生物医学期刊文献数据库)

Search time: 26 July 2010

|  | **Results** |  | **Search Strategy** |
| --- | --- | --- | --- |
| 1 | 16 |  | ((临床试验/FLD=关键词 OR 对照试验/FLD=关键词 OR 随机/FLD=关键词) AND (冠心病/FLD=题名,关键词 OR 心肌梗死/FLD=题名,关键词 OR 心肌梗塞/FLD=题名,关键词 OR 心绞痛/FLD=题名,关键词) AND (丹参/FLD=题名,关键词 OR 葛根/FLD=题名,关键词 OR 细辛/FLD=题名,关键词 OR 红花/FLD=题名,关键词 OR 三七/FLD=题名,关键词 OR 刺五加/FLD=题名,关键词 OR 毛冬青/FLD=题名,关键词 OR 益母草/FLD=题名,关键词 OR 延胡索/FLD=题名,关键词 OR 赤芍/FLD=题名,关键词 OR 蒲黄/FLD=题名,关键词 OR 淫羊藿/FLD=题名,关键词 OR 瓜蒌/FLD=题名,关键词 OR 防己/FLD=题名,关键词 OR 灵芝/FLD=题名,关键词 OR 山楂/FLD=题名,关键词 OR 当归/FLD=题名,关键词 OR 川穹/FLD=题名,关键词 OR 麝香/FLD=题名,关键词 OR 苏合香/FLD=题名,关键词 OR 灯盏花/FLD=题名,关键词 OR 银杏叶/FLD=题名,关键词 OR 阿槐酸钠/FLD=题名,关键词 OR 速效救心/FLD=题名,关键词 OR 冠心苏合/FLD=题名,关键词 OR 苏心丸/FLD=题名,关键词 OR 益心丸/FLD=题名,关键词 OR 心灵丸/FLD=题名,关键词 OR 心宝/FLD=题名,关键词 OR 右归/FLD=题名,关键词 OR 护心丹/FLD=题名,关键词 OR 救心丹/FLD=题名,关键词 OR 环心丹/FLD=题名,关键词 OR 活心丹/FLD=题名,关键词 OR 苏冰滴丸/FLD=题名,关键词 OR 冠心膏/FLD=题名,关键词)) SUB (#文献类型=Z OR #文献类型=J OR #文献类型=W OR #文献类型=Y OR 动物/FLD=关键词 OR 浅谈/FLD=题名 OR 浅析/FLD=题名 OR 初探/FLD=题名 OR 新用/FLD=题名 OR 巧用/FLD=题名 OR 漫谈/FLD=题名 OR 治验/FLD=题名 OR 体会/FLD=题名 OR 举验/FLD=题名 OR 举隅/FLD=题名 OR 举要/FLD=题名 OR 案例/FLD=题名 OR 刍议/FLD=题名 OR 经验/FLD=题名 OR 验案/FLD=题名 OR 配伍分析/FLD=题名 OR 概述/FLD=题名) |
| 2 | 926 |  | ((随机/FLD=摘要 AND 对照/FLD=摘要) AND (冠心病/FLD=题名,关键词 OR 心肌梗死/FLD=题名,关键词 OR 心肌梗塞/FLD=题名,关键词 OR 心绞痛/FLD=题名,关键词) AND (丹参/FLD=题名,关键词 OR 葛根/FLD=题名,关键词 OR 细辛/FLD=题名,关键词 OR 红花/FLD=题名,关键词 OR 三七/FLD=题名,关键词 OR 刺五加/FLD=题名,关键词 OR 毛冬青/FLD=题名,关键词 OR 益母草/FLD=题名,关键词 OR 延胡索/FLD=题名,关键词 OR 赤芍/FLD=题名,关键词 OR 蒲黄/FLD=题名,关键词 OR 淫羊藿/FLD=题名,关键词 OR 瓜蒌/FLD=题名,关键词 OR 防己/FLD=题名,关键词 OR 灵芝/FLD=题名,关键词 OR 山楂/FLD=题名,关键词 OR 当归/FLD=题名,关键词 OR 川穹/FLD=题名,关键词 OR 麝香/FLD=题名,关键词 OR 苏合香/FLD=题名,关键词 OR 灯盏花/FLD=题名,关键词 OR 银杏叶/FLD=题名,关键词 OR 阿槐酸钠/FLD=题名,关键词 OR 速效救心/FLD=题名,关键词 OR 冠心苏合/FLD=题名,关键词 OR 苏心丸/FLD=题名,关键词 OR 益心丸/FLD=题名,关键词 OR 心灵丸/FLD=题名,关键词 OR 心宝/FLD=题名,关键词 OR 右归/FLD=题名,关键词 OR 护心丹/FLD=题名,关键词 OR 救心丹/FLD=题名,关键词 OR 环心丹/FLD=题名,关键词 OR 活心丹/FLD=题名,关键词 OR 苏冰滴丸/FLD=题名,关键词 OR 冠心膏/FLD=题名,关键词)) SUB (#文献类型=Z OR #文献类型=J OR #文献类型=W OR #文献类型=Y OR 动物/FLD=关键词 OR 浅谈/FLD=题名 OR 浅析/FLD=题名 OR 初探/FLD=题名 OR 新用/FLD=题名 OR 巧用/FLD=题名 OR 漫谈/FLD=题名 OR 治验/FLD=题名 OR 体会/FLD=题名 OR 举验/FLD=题名 OR 举隅/FLD=题名 OR 举要/FLD=题名 OR 案例/FLD=题名 OR 刍议/FLD=题名 OR 经验/FLD=题名 OR 验案/FLD=题名 OR 配伍分析/FLD=题名 OR 概述/FLD=题名) |
| 3 | 6 |  | ((临床试验/FLD=关键词 OR 对照试验/FLD=关键词 OR 随机/FLD=关键词) AND (冠心病/FLD=题名,关键词 OR 心肌梗死/FLD=题名,关键词 OR 心肌梗塞/FLD=题名,关键词 OR 心绞痛/FLD=题名,关键词) AND (心绞痛宁膏/FLD=题名,关键词 OR 救心油/FLD=题名,关键词 OR 养心氏片/FLD=题名,关键词 OR 心可舒/FLD=题名,关键词 OR 稳心颗粒/FLD=题名,关键词 OR 丹七片/FLD=题名,关键词 OR 丹蒌片/FLD=题名,关键词 OR 心血宁/FLD=题名,关键词 OR 通心络/FLD=题名,关键词 OR 心脉通/FLD=题名,关键词 OR 脑心通/FLD=题名,关键词 OR 益心舒/FLD=题名,关键词 OR 心可宁/FLD=题名,关键词 OR 诺迪康/FLD=题名,关键词 OR 血脉康/FLD=题名,关键词 OR 心血康/FLD=题名,关键词 OR 珠麝消栓/FLD=题名,关键词 OR 心脑舒通/FLD=题名,关键词 OR 血府逐瘀/FLD=题名,关键词 OR 心元胶囊/FLD=题名,关键词 OR 通冠胶囊/FLD=题名,关键词 OR 活血通脉胶囊/FLD=题名,关键词 OR 补心气口服液/FLD=题名,关键词 OR 心通口服液/FLD=题名,关键词 OR 舒心口服液/FLD=题名,关键词 OR 滋心阴口服液/FLD=题名,关键词 OR 参芍/FLD=题名,关键词 OR 生脉/FLD=题名,关键词 OR 血塞通/FLD=题名,关键词 OR 血栓通/FLD=题名,关键词 OR 脉络宁/FLD=题名,关键词 OR 舒血宁/FLD=题名,关键词 OR 疏血通/FLD=题名,关键词 OR 冠心宁/FLD=题名,关键词 OR 脉普林/FLD=题名,关键词 OR 醒脑静/FLD=题名,关键词 OR 清开灵/FLD=题名,关键词 OR 参附注射液/FLD=题名,关键词 OR 参麦注射液/FLD=题名,关键词 OR 地龙注射液/FLD=题名,关键词 OR 黄芪注射液/FLD=题名,关键词 OR 宽胸气雾剂/FLD=题名,关键词 OR 心痛气雾剂/FLD=题名,关键词)) SUB (#文献类型=Z OR #文献类型=J OR #文献类型=W OR #文献类型=Y OR 动物/FLD=关键词 OR 浅谈/FLD=题名 OR 浅析/FLD=题名 OR 初探/FLD=题名 OR 新用/FLD=题名 OR 巧用/FLD=题名 OR 漫谈/FLD=题名 OR 治验/FLD=题名 OR 体会/FLD=题名 OR 举验/FLD=题名 OR 举隅/FLD=题名 OR 举要/FLD=题名 OR 案例/FLD=题名 OR 刍议/FLD=题名 OR 经验/FLD=题名 OR 验案/FLD=题名 OR 配伍分析/FLD=题名 OR 概述/FLD=题名) |
| 4 | 981 |  | ((随机/FLD=摘要 AND 对照/FLD=摘要) AND (冠心病/FLD=题名,关键词 OR 心肌梗死/FLD=题名,关键词 OR 心肌梗塞/FLD=题名,关键词 OR 心绞痛/FLD=题名,关键词) AND (心绞痛宁膏/FLD=题名,关键词 OR 救心油/FLD=题名,关键词 OR 养心氏片/FLD=题名,关键词 OR 心可舒/FLD=题名,关键词 OR 稳心颗粒/FLD=题名,关键词 OR 丹七片/FLD=题名,关键词 OR 丹蒌片/FLD=题名,关键词 OR 心血宁/FLD=题名,关键词 OR 通心络/FLD=题名,关键词 OR 心脉通/FLD=题名,关键词 OR 脑心通/FLD=题名,关键词 OR 益心舒/FLD=题名,关键词 OR 心可宁/FLD=题名,关键词 OR 诺迪康/FLD=题名,关键词 OR 血脉康/FLD=题名,关键词 OR 心血康/FLD=题名,关键词 OR 珠麝消栓/FLD=题名,关键词 OR 心脑舒通/FLD=题名,关键词 OR 血府逐瘀/FLD=题名,关键词 OR 心元胶囊/FLD=题名,关键词 OR 通冠胶囊/FLD=题名,关键词 OR 活血通脉胶囊/FLD=题名,关键词 OR 补心气口服液/FLD=题名,关键词 OR 心通口服液/FLD=题名,关键词 OR 舒心口服液/FLD=题名,关键词 OR 滋心阴口服液/FLD=题名,关键词 OR 参芍/FLD=题名,关键词 OR 生脉/FLD=题名,关键词 OR 血塞通/FLD=题名,关键词 OR 血栓通/FLD=题名,关键词 OR 脉络宁/FLD=题名,关键词 OR 舒血宁/FLD=题名,关键词 OR 疏血通/FLD=题名,关键词 OR 冠心宁/FLD=题名,关键词 OR 脉普林/FLD=题名,关键词 OR 醒脑静/FLD=题名,关键词 OR 清开灵/FLD=题名,关键词 OR 参附注射液/FLD=题名,关键词 OR 参麦注射液/FLD=题名,关键词 OR 地龙注射液/FLD=题名,关键词 OR 黄芪注射液/FLD=题名,关键词 OR 宽胸气雾剂/FLD=题名,关键词 OR 心痛气雾剂/FLD=题名,关键词 OR 补心气口服液/FLD=题名,关键词 OR 心通口服液/FLD=题名,关键词 OR 舒心口服液/FLD=题名,关键词 OR 滋心阴口服液/FLD=题名,关键词 OR 参芍/FLD=题名,关键词 OR 生脉/FLD=题名,关键词 OR 血塞通/FLD=题名,关键词 OR 血栓通/FLD=题名,关键词 OR 脉络宁/FLD=题名,关键词 OR 舒血宁/FLD=题名,关键词 OR 疏血通/FLD=题名,关键词 OR 冠心宁/FLD=题名,关键词 OR 脉普林/FLD=题名,关键词 OR 醒脑静/FLD=题名,关键词 OR 清开灵/FLD=题名,关键词 OR 参附注射液/FLD=题名,关键词 OR 参麦注射液/FLD=题名,关键词 OR 地龙注射液/FLD=题名,关键词 OR 黄芪注射液/FLD=题名,关键词 OR 宽胸气雾剂/FLD=题名,关键词 OR 心痛气雾剂/FLD=题名,关键词)) SUB (#文献类型=Z OR #文献类型=J OR #文献类型=W OR #文献类型=Y OR 动物/FLD=关键词 OR 浅谈/FLD=题名 OR 浅析/FLD=题名 OR 初探/FLD=题名 OR 新用/FLD=题名 OR 巧用/FLD=题名 OR 漫谈/FLD=题名 OR 治验/FLD=题名 OR 体会/FLD=题名 OR 举验/FLD=题名 OR 举隅/FLD=题名 OR 举要/FLD=题名 OR 案例/FLD=题名 OR 刍议/FLD=题名 OR 经验/FLD=题名 OR 验案/FLD=题名 OR 配伍分析/FLD=题名 OR 概述/FLD=题名) |
| 5 | 9 |  | ((临床试验/FLD=关键词 OR 对照试验/FLD=关键词 OR 随机/FLD=关键词) AND (冠心病/FLD=题名,关键词 OR 心肌梗死/FLD=题名,关键词 OR 心肌梗塞/FLD=题名,关键词 OR 心绞痛/FLD=题名,关键词) AND (中药/FLD=关键词 OR 中草药/FLD=关键词 OR 中成药/FLD=关键词 OR 中西医结合/FLD=关键词)) SUB (#文献类型=Z OR #文献类型=J OR #文献类型=W OR #文献类型=Y OR 动物/FLD=关键词 OR 浅谈/FLD=题名 OR 浅析/FLD=题名 OR 初探/FLD=题名 OR 新用/FLD=题名 OR 巧用/FLD=题名 OR 漫谈/FLD=题名 OR 治验/FLD=题名 OR 体会/FLD=题名 OR 举验/FLD=题名 OR 举隅/FLD=题名 OR 举要/FLD=题名 OR 案例/FLD=题名 OR 刍议/FLD=题名 OR 经验/FLD=题名 OR 验案/FLD=题名 OR 配伍分析/FLD=题名 OR 概述/FLD=题名) |
| 6 | 341 |  | ((随机/FLD=摘要 AND 对照/FLD=摘要) AND (冠心病/FLD=题名,关键词 OR 心肌梗死/FLD=题名,关键词 OR 心肌梗塞/FLD=题名,关键词 OR 心绞痛/FLD=题名,关键词) AND (中药/FLD=关键词 OR 中草药/FLD=关键词 OR 中成药/FLD=关键词 OR 中西医结合/FLD=关键词)) SUB (#文献类型=Z OR #文献类型=J OR #文献类型=W OR #文献类型=Y OR 动物/FLD=关键词 OR 浅谈/FLD=题名 OR 浅析/FLD=题名 OR 初探/FLD=题名 OR 新用/FLD=题名 OR 巧用/FLD=题名 OR 漫谈/FLD=题名 OR 治验/FLD=题名 OR 体会/FLD=题名 OR 举验/FLD=题名 OR 举隅/FLD=题名 OR 举要/FLD=题名 OR 案例/FLD=题名 OR 刍议/FLD=题名 OR 经验/FLD=题名 OR 验案/FLD=题名 OR 配伍分析/FLD=题名 OR 概述/FLD=题名) |

TCMLARS (Traditional Chinese Medical Literature Analysis and Retrieval System, 中国中医药期刊文献数据库)

Search time: 26 July 2010

|  | **Results** |  | **Search Strategy** |
| --- | --- | --- | --- |
| 1 | 4117 |  | ((( (WXMES=心肌梗塞\/中医药疗法) OR (WXMES=心肌梗塞\/中西医结合疗法) ) OR ( WXMES = ('心肌顿抑\/中医药疗法','心肌顿抑\/中西医结合疗法','休克，心原性\/中医药疗法','休克，心原性\/中西医结合疗法','心肌顿抑\/中医药疗法','心肌顿抑\/中西医结合疗法','无复流现象\/中医药疗法','无复流现象\/中西医结合疗法','休克，心原性\/中医药疗法','休克，心原性\/中西医结合疗法'))) OR (( (WXMES=心绞痛\/中医药疗法) OR (WXMES=心绞痛\/中西医结合疗法) ) OR ( WXMES = ('心绞痛，不稳定型\/中医药疗法','心绞痛，不稳定型\/中西医结合疗法','心绞痛，变异型\/中医药疗法','心绞痛，变异型\/中西医结合疗法','微血管性心绞痛\/中医药疗法','微血管性心绞痛\/中西医结合疗法','心绞痛，不稳定型\/中医药疗法','心绞痛，不稳定型\/中西医结合疗法','心绞痛，变异型\/中医药疗法','心绞痛，变异型\/中西医结合疗法','微血管性心绞痛\/中医药疗法','微血管性心绞痛\/中西医结合疗法','心绞痛，不稳定型\/中医药疗法','心绞痛，不稳定型\/中西医结合疗法','心绞痛，变异型\/中医药疗法','心绞痛，变异型\/中西医结合疗法'))) AND ( WXTYP=随机对照试验 OR WXTAG=随机对照试验 OR WXMES=随机对照试验) AND ( WXTAG=人类 OR WXTYP=人类 OR WXMES=人类) NOT ( WXTAG=动物 OR WXTYP=动物 OR WXMES=动物)) NOT (WXTIC=浅谈 or 浅析 or 初探 or 新用 or 巧用 or 漫谈 or 治验 or 体会 or 举验 or 举隅 or 举要 or 案例 or 刍议 or 经验 or 验案 or 配伍分析 or 概述) |

**Appendix 2: Detailed Risk of Bias Assessment Results**

| First author | Year | Risk of bias  for randomization | Risk of bias for allocation concealment | Risk of bias for blinding | Risk of bias for incomplete data | Risk of bias for selective outcome reporting | Risk bias for other bias |
| --- | --- | --- | --- | --- | --- | --- | --- |
|  |  |  |  |  |  |  |  |
| CHD Group^21^ | 1981 | High | High | Low | Low | Low | Low |
| Kou WR^22^ | 1983 | High | High | Low | Low | Low | Low |
| Chen KJ^23^ | 1984 | Uncertain | High | Low | Low | Low | Low |
| Liang SL^24^ | 1989 | High | High | Low | Low | Low | Low |
| Xia XQ^25^ | 1993 | Uncertain | High | Low | Low | Low | Low |
| Li CK^26^ | 1994 | High | High | Low | Low | Low | Low |
| Li SQ^27^ | 1994 | Uncertain | High | Low | Low | Low | Low |
| Yang GM^28^ | 1997 | High | High | Low | Low | Low | Low |
| Zhang RJ^29^ | 1998 | Uncertain | High | Low | Low | Low | Low |
| Guo SP^30^ | 1999 | Uncertain | High | Low | Low | Low | Low |
| Li GQ^31^ | 1999 | High | High | Low | Low | Low | Low |
| Zhang DJ^32^ | 1999 | Uncertain | High | Low | Low | Low | Low |
| Guo SP^33^ | 2000 | High | High | Low | Low | Low | Low |
| Han GJ^34^ | 2000 | Uncertain | High | Low | Low | Low | Low |
| Li J^35^ | 2000 | Uncertain | High | Low | Low | Low | Low |
| Li QZ(a)^36^ | 2000 | High | High | Low | Low | Low | Low |
| Li QZ(b)^36^ | 2000 | High | High | Low | Low | Low | Low |
| Lu JQ^37^ | 2000 | Uncertain | High | Low | Low | Low | Low |
| Yin KC^38^ | 2000 | Uncertain | High | Low | Low | Low | Low |
| Wu G^39^ | 2002 | Uncertain | High | Low | Low | Low | Low |
| Bai YK ^40^ | 2002 | Low | Low | Low | Low | Low | Low |
| Shi JL^41^ | 2002 | Low | High | Low | Low | Low | Low |
| Guan Y^42^ | 2003 | Uncertain | High | Low | Low | Low | Low |
| Zhang SF^43^ | 2003 | Uncertain | High | Low | Low | Low | Low |
| Han GJ^44^ | 2004 | Uncertain | High | Low | Low | Low | Low |
| Li XF^45^ | 2004 | High | High | Low | Low | Low | Low |
| Liu SK^46^ | 2004 | Uncertain | High | Low | Low | Low | High^△^ |
| Yang GZ^47^ | 2004 | Uncertain | High | Low | Low | Low | Low |
| Chen LJ ^48^ | 2005 | Uncertain | High | Low | Low | Low | Low |
| Deng YL^49^ | 2005 | Uncertain | High | Low | Low | Low | Low |
| He YH^50^ | 2005 | Uncertain | High | Low | Low | Low | Low |
| Li HT^51^ | 2005 | Uncertain | High | Low | Low | Low | Low |
| Liu YQ^52*^ | 2005 | Uncertain | High | Low | Low | Low | Low |
| Miao YM^53^ | 2005 | Uncertain | High | Low | Low | Low | Low |
| Yang Y^54^ | 2005 | Low | High | Low | Low | Low | Low |
| Ding L^55^ | 2006 | Uncertain | High | Low | Low | Low | High^△^ |
| Du BM(a)^56^ | 2006 | Uncertain | Uncertain | Low | Low | Low | Low |
| Du BM(b)^56^ | 2006 | Uncertain | Uncertain | Low | Low | Low | Low |
| Li ZE^57^ | 2006 | High | High | Low | Low | Low | Low |
| Ma LB^58^ | 2006 | Low | High | Low | Low | Low | Low |
| Qi H^59^ | 2006 | Uncertain | High | Low | Low | Low | High^△^ |
| Shen YX^60^ | 2006 | Low | High | Low | Low | Low | Low |
| Wang SL^61^ | 2006 | Uncertain | High | Low | Low | Low | High^△^ |
| Wei XC^62^ | 2006 | Uncertain | High | Low | Low | Low | Low |
| Wu JG^63^ | 2006 | High | High | Low | Low | Low | Low |
| Yang JW^64^ | 2006 | Low | Low | Low | Low | Low | Low |
| Chen H^65^ | 2007 | Low | High | Low | Low | Low | Low |
| Li Z^66^ | 2007 | Uncertain | High | Low | Low | Low | Low |
| Liang C^67^* | 2007 | High | High | Low | Low | Low | Low |
| Pan G ^68^ | 2007 | Uncertain | High | Low | Low | Low | Low |
| Qu YZ^69^ | 2007 | Uncertain | High | Low | Low | Low | Low |
| Ding YB^70^ | 2008 | Uncertain | High | Low | Low | Low | Low |
| Lan J^71^ | 2008 | Uncertain | High | Low | Low | Low | High^△^ |
| Yu CJ^72^ | 2008 | Uncertain | High | Low | Low | Low | Low |
| Yu T^73^ | 2008 | Uncertain | High | Low | Low | Low | Low |
| Zhang J^74^ | 2008 | Uncertain | High | Low | High | Low | Low |
| Gao ZQ^75^ | 2009 | Uncertain | High | Low | Low | Low | Low |
| Lin H^76^ | 2009 | Uncertain | High | Low | Low | Low | Low |
| Liu PF^77^ | 2009 | Uncertain | High | Low | Low | Low | High^△^ |
| Song XH^78^ | 2009 | Uncertain | High | Low | Low | Low | Low |
| Yuan XH^79^ | 2009 | Uncertain | High | Low | Low | Low | Low |
| Zhao QH^80^ | 2009 | Uncertain | High | Low | Low | Low | Low |
| Zuo Y^81^ | 2009 | Uncertain | High | Low | Low | Low | Low |
| Guo HL^82^ | 2010 | Uncertain | High | Low | High | High | Low |
| Xu Q^83^* | 2010 | Uncertain | High | Low | Low | Low | Low |

* Two RCTs were reported in each of the publication. Similar methodologies were used for both RCTs.

^△^ Reasons for risk of other biases:

Ding L^55^ 2006: inconsistent description on the fatality rate in both CHM+BM group and BM only group;

Qi H^59^ 2006: Side effects and adverse reactions were described in CHM+BM group but not in BM only group;

Wang SL^61^ 2006: inconsistent reporting on sample size;

Lan J^71^ 2008: inconsistent reporting on the number of patients in the control group;

Liu PF^77^ 2009: no reasons were provided for losses to follow up.
